# Supplementary material for: SLIDE—Novel Approach to Apocrine Sweat Sampling for Lipid Profiling in Healthy Individuals
Source: Int J Mol Sci. 2021 Jul 28;22(15):8054. doi: 10.3390/ijms22158054 (PMC8348598; doi:10.3390/ijms22158054)

Article – Supplementary material

SLIDE – Novel Approach to Apocrine Sweat Sampling for Lipid Profiling in Healthy Individuals

Aleš Kvasnička [1†], David Friedecký [1,2†*], Alena Tichá [3], Radomír Hyšpler [3], Hana Janečková [2], Radana Brumarová [1], Lukáš Najdekr [1], Zdeněk Zadák [4]

[1] Faculty of Medicine and Dentistry, Palacký University Olomouc, Czechia; ales.kvasnicka01@upol.cz (A.K.), radana.karlikova@gmail.com (R.B.), lukas.najdekr@gmail.com (L.N.)

[2] Laboratory for Inherited Metabolic Disorders, Department of Clinical Chemistry, University Hospital, Olomouc, Czechia; david.friedecky@upol.cz (D.F.); janeckovah@gmail.com (H.J.)

[3] Department of Clinical Biochemistry and Diagnostics and Osteocenter, University Hospital Hradec Králové, Sokolská 581, Hradec Králové, Czechia; alena.ticha@fnhk.cz (A.T.); radomir.hyspler@fnhk.cz (R.H.)

[4] Department of Research and Development, University Hospital Hradec Králové, Sokolská 581, Hradec Králové, Czechia; zdenek.zadak@fnhk.cz (Z.Z.)

* Correspondence: david.friedecky@upol.cz; Tel.: +420 604 871 961

† These authors contributed equal work to this project.

Sample description

Table S1. Anonymized sample information. ID of samples consisting of order of participant (first letter; A-J), day of sampling (second number; 1-3) and side of armpit (third letter, left – L and right – R)).

Participant	Sample ID	Sex	Year of birth
To-Ba	A1L / A1R / A2L / A2R / A3L / A3R	M	1999
Kl-Pi	B1L / B1R / B2L / B2R / B3L / B3R	F	1999
An-Cv	C1L / C1R / C2L / C2R / C3L / C3R	F	1999
Ta-Va	D1L / D1R / D2L / D2R / D3L / D3R	F	2000
Kr-Bu	E1L / E1R / E2L / E2R / E3L / E3R	F	1999
Ma-Kr	F1L / F1R / F2L / F2R / F3L / F3R	F	2000
An-Po	G1L / G1R / G2L / G2R / G3L / G3R	F	2000
Ad-Ne	H1L / H1R / H2L / H2R / H3L / H3R	M	2000
Zd-Za	I1L / I1R / I2L / I2R / I3L / I3R	M	1937
Ma-Mz	J1L / J1R / J2L / J2R / J3L / J3R	M	1987

Pseudotargeted lipidomic analysis

Table S2. Pseudotargeted lipidomic method MRM specifications for lipid classes, which were selected for the final method; FA – fatty acyl; HG – head group; LCB – long chain base.

Class	Precursor ion adduct	MRM (type, m/z)
CE	$[M+NH4]^{+}$	$[C27H44; 369]^{+}$
FA	$[M-H]^{-}$	$[FA+O]^{-}$
LPC	$[M+H]^{+}$	$[HG(PC; 184)]^{+}$
LPE	$[M+H]^{+}$	$[-HG(PE; 141)]^{+}$
Cer	$[M+H]^{+}$	$[LCB(-H3O2)]^{+}$
HexCer	$[M+H]^{+}$	$[LCB(-H3O2)]^{+}$
PE	$[M+H]^{+}$	$[-HG(PE; 141)]^{+}$
SM	$[M+H]^{+}$	$[HG(PC; 184)]^{+}$
PC	$[M+H]^{+}$	$[HG(PC; 184)]^{+}$
PI	$[M-H]^{-}$	$[HG(PI; 241)]^{-}$
PS	$[M-H]^{-}$	$[-(C3H5NO2; 87)]^{-}$
PEO	$[M+H]^{+}$	$[-FA+(C3H5O2)]^{+}$
PCO	$[M+H]^{+}$	$[HG(PC; 184)]^{+}$
TG	$[M+NH4]^{+}$	$[-FA+(HO)-(NH3)]^{+}$
DG	$[M+NH4]^{+}$	$[-FA+(HO)-(NH3)]^{+}$

Table S3. Concentration of each matrix-matched standard calibration series created by dilution of QC sample by STD mix (prepared using isotopically-labeled SPLASH® LIPIDOMIX® Mass Spec Standard mixture with the addition of two more deuterated standards: arachidonic acid (d8) and ceramide d18:1d7/15:0).

Lipid Class	STD	Concentration (ng/ml)					
		**Concentration of 10000x diluted** STD mix [ng/mL]	**Concentration of 1000x diluted** STD mix [ng/mL]	**Concentration of 500x diluted** STD mix [ng/mL]	**Concentration of 100x diluted** STD mix [ng/mL]	**Concentration of 50x diluted** STD mix [ng/mL]	**Concentration of 10x diluted** STD mix [ng/mL]
CE	CE (18:1-d7)	29.62	296.19	592.38	2961.90	5923.80	29619.00
PC	PC (15:0/18:1-d7)	13.55	135.54	271.08	1355.40	2710.80	13554.00
Chol	Cholesterol (d7)	8.86	88.56	177.12	885.60	1771.20	8856.00
TAG	TG (15:0/18:1-d7/15:0)	4.75	47.52	95.04	475.20	950.40	4752.00
SM	SM (d18:1/18:1-d9)	2.66	26.64	53.28	266.40	532.80	2664.00
PG	PG (15:0/18:1-d7)	2.33	23.34	46.68	233.38	466.76	2333.82
LPC	LPC (18:1-d7)	2.14	21.42	42.84	214.20	428.40	2142.00
DAG	DG (15:0/18:1-d7)	0.79	7.92	15.84	79.20	158.40	792.00
PI	PI (15:0/18:1-d7)	0.75	7.50	14.99	74.96	149.92	749.61
PA	PA (15:0/18:1-d7)	0.60	6.01	12.02	60.12	120.24	601.20
PE	PE (15:0/18:1-d7)	0.48	4.77	9.54	47.70	95.40	477.00
LPE	LPE (18:1-d7)	0.44	4.41	8.82	44.10	88.20	441.00
PS	PS (15:0/18:1-d7)	0.34	3.41	6.82	34.11	68.21	341.06
MAG	MG (18:1-d7)	0.16	1.62	3.24	16.20	32.40	162.00
Cer	Cer (d18:1(d7)/18:0)	5.00	50.00	100.00	500.00	1000.00	5000.00
FA	Arachidonic acid: FA 20:4-d8	5.00	50.00	100.00	500.00	1000.00	5000.00

Table S4. Calibrations series and quantitation specifications of linear response (y=ax+b). Only the lipid classes selected for the final method were considered. Individual standards (STD) were used for quantification of multiple lipid classes of similar structural origin (Class column).

STD	Class	a	b	R^2	Weighting
CE (18:1-d7)	CE	7.48E+00	4.99E+03	0.9991	$1/y^2$
Cer (d18:1(d7)/18:0)	Cer, HexCer	6.11E+03	3.67E+03	0.9980	$1/y^2$
DG (15:0/18:1-d7)	DG	1.17E+03	6.68E+01	0.9819	$1/y^2$
Arachidonic acid: FA 20:4-d8	FA	6.21E+03	2.75E+05	0.9993	1/y
LPC (18:1-d7)	LPC	9.19E+04	6.62E+04	0.9998	1/y
LPE (18:1-d7)	LPE	1.86E+04	2.19E+03	0.9989	$1/y^2$
PC (15:0/18:1-d7)	PC, PCO	9.32E+04	7.58E+04	0.9996	1/y
PE (15:0/18:1-d7)	PE, PEO	2.17E+04	6.02E+03	0.9986	$1/y^2$
PI (15:0/18:1-d7)	PI	1.58E+03	4.48E+02	0.9932	$1/y^2$
PS (15:0/18:1-d7)	PS	1.70E+03	1.30E+02	0.9992	$1/y^2$
SM (d18:1/18:1-d9)	SM	4.07E+04	4.64E+04	0.9988	$1/y^2$
TG (15:0/18:1-d7/15:0)	TG	1.28E+04	2.25E+04	0.9848	$1/y^2$

Figure S1. Lipid patterns plotted for each lipid class. Charts are plotted as *m/z* value on the y-axis and retention time in minutes on the x-axis. Saturation equals to the number of double bonds. Lipid pattern plots were used to correctly assign lipid annotations and to annulate potential misidentifications. Few shifts can be observed for example in ceramides with 1 double bond, which is occurring due to different long-chain base composition (d16:1, d18:1, or d20:1), or in the case of PC-O species where both ether (plasmanyl) and vinyl ether (plasmenyl) species are considered in one plot. Lipid isomers with different acyl-composition can also differ in retention time resulting in non-absolute linear/quadratic pattern curve observed (for example in the case of PC 36:4).

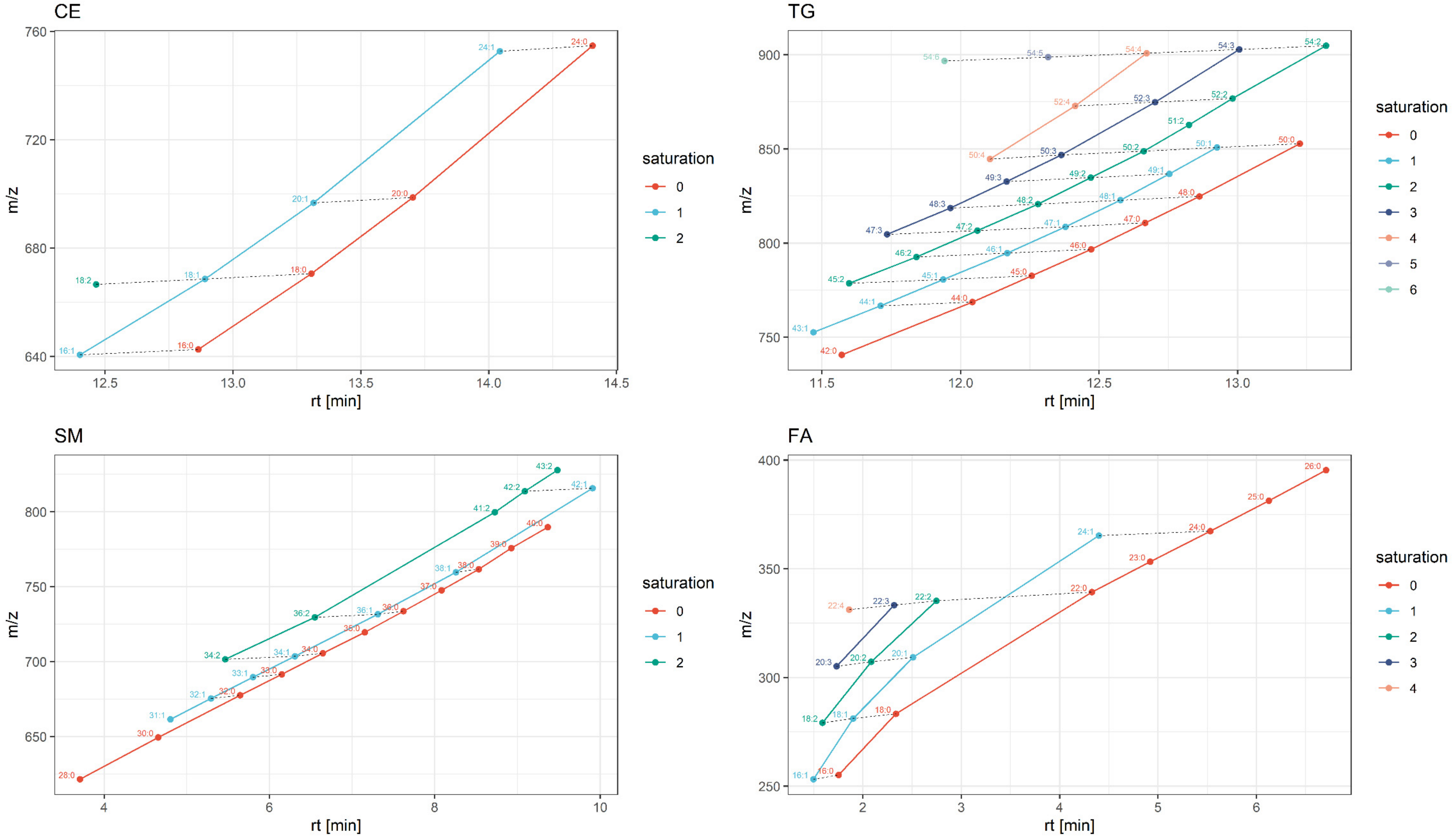

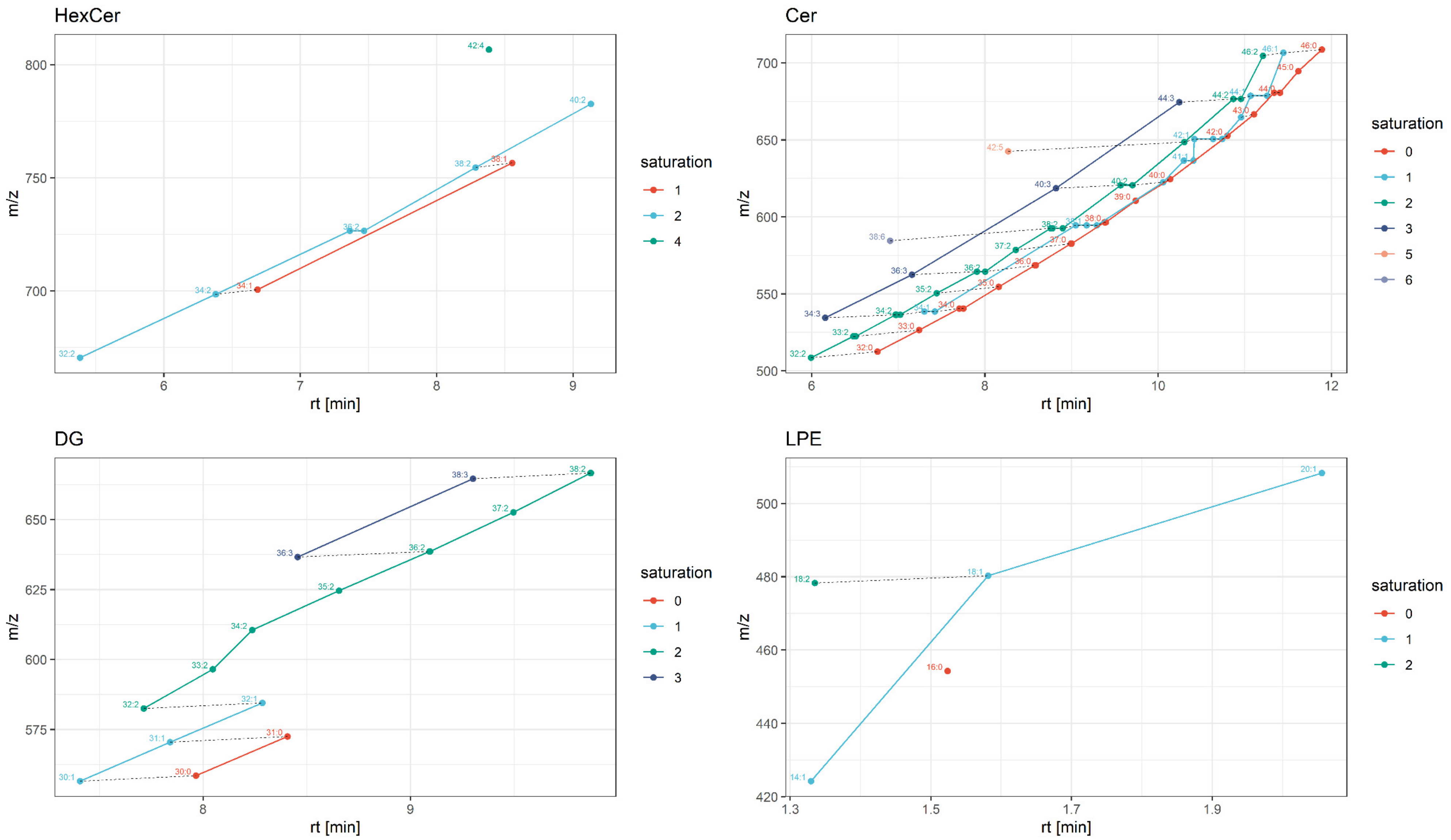
HexCer
m/z
rt [min]
saturation
1
2
4
Cer
m/z
rt [min]
saturation
0
1
2
3
5
6
DG
m/z
rt [min]
saturation
0
1
2
3
LPE
m/z
rt [min]
saturation
0
1
2

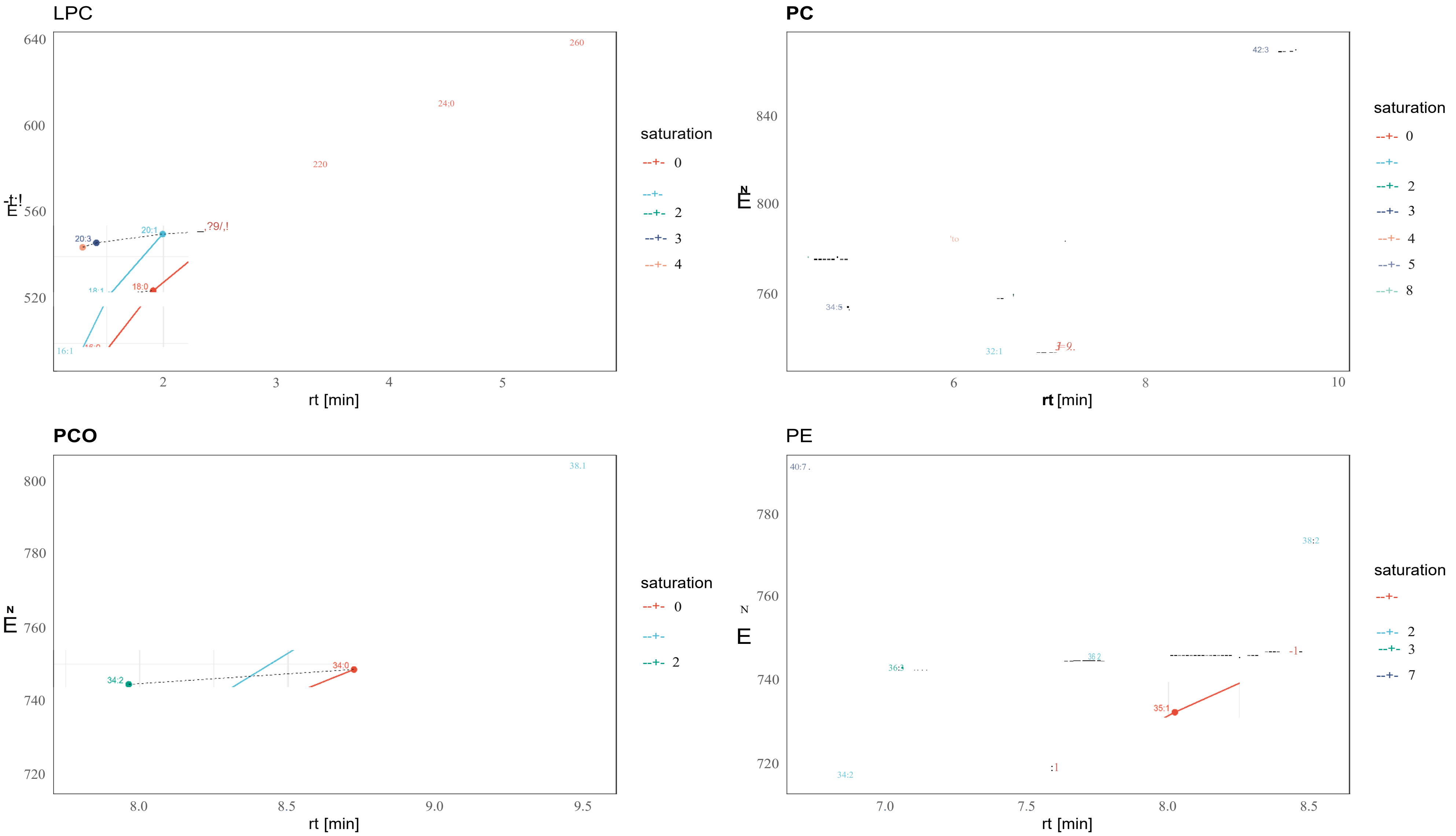
LPC
640
600
560
520
2
3
4
5
rt [min]
260
24:0
220
20:3
20:1
18:0
16:1
saturation
0
2
3
4
PC
840
800
760
6
8
10
rt [min]
42:3
34:5
32:1
saturation
0
2
3
4
5
8
PCO
800
780
760
740
720
8.0
8.5
9.0
9.5
rt [min]
38:1
34:2
34:0
saturation
0
2
PE
780
760
740
720
7.0
7.5
8.0
8.5
rt [min]
40:7
38:2
36:3
35:1
34:2
saturation
2
3
7

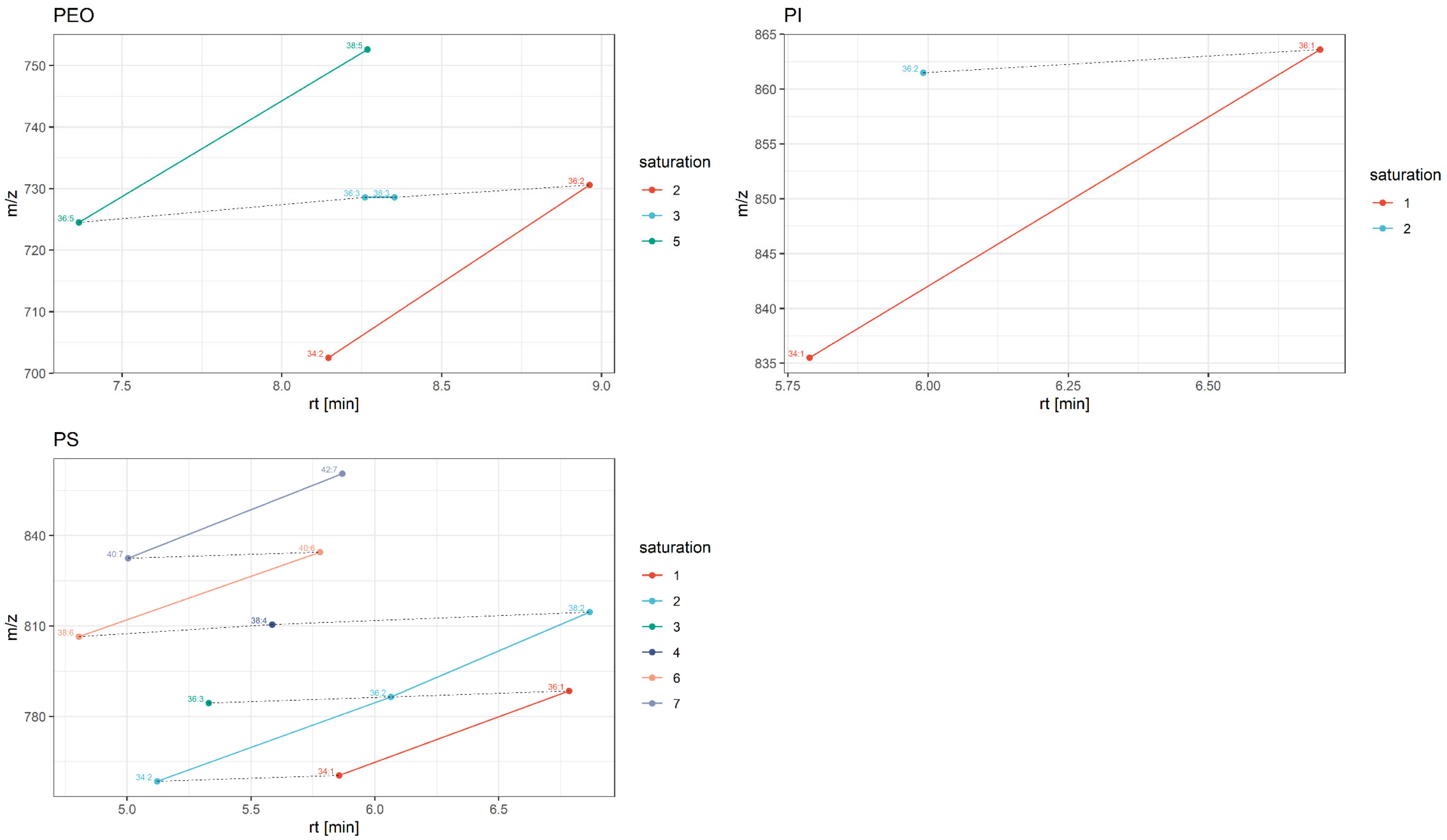

PEO
m/z
rt [min]
750
740
730
720
710
700
7.5
8.0
8.5
9.0
38:5
36:3
38:3
36:2
36:5
34:2
saturation
2
3
5
PI
m/z
rt [min]
865
860
855
850
845
840
835
5.75
6.00
6.25
6.50
36:1
36:2
34:1
saturation
1
2
PS
m/z
rt [min]
840
810
780
5.0
5.5
6.0
6.5
42:7
40:7
40:6
38:2
38:4
38:6
36:3
36:2
36:1
34:2
34:1
saturation
1
2
3
4
6
7

Figure S2. Coelution pattern for PC, PCO and SM classes. PC, SM and PCO lipid species all provide the same fragment – phosphatidylcholine head group [HG(PC; 184)] $^{+}$, therefore potential coelution needs to be monitored. A close coelution of PC 32:1 and SM 36:2 has been observed, however given the fact of the 3 *m/z* unit difference between these two coeluting species, this does not cause a problem (when one of these species is not dramatically more abundant, which did not occur in our case). Coelution of PCO 32:0 and 32:1 is caused by two structural variants (plasmanyl – ether and plasmenyl – vinylether type bonds).

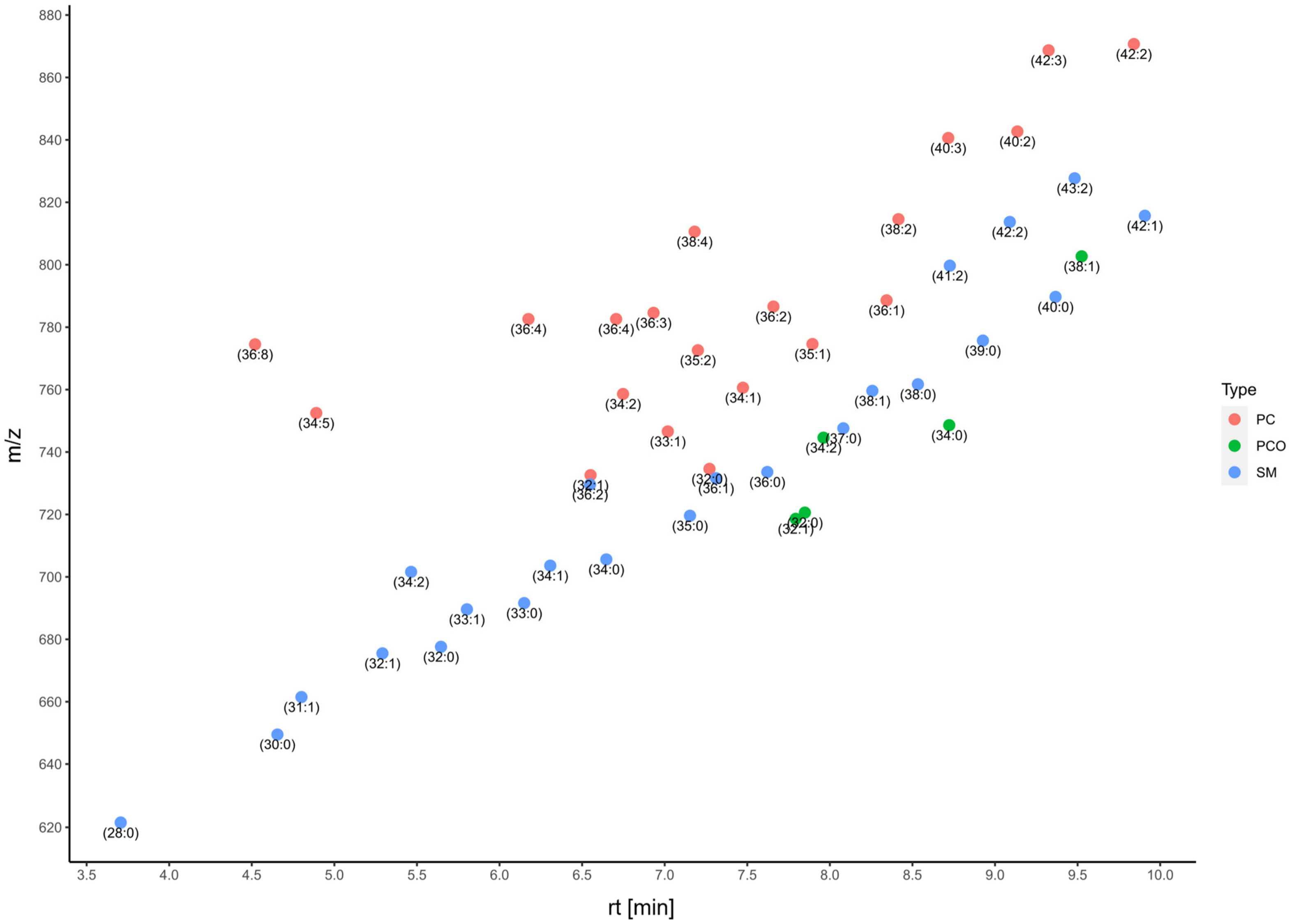

Figure S3. Injection volume experiment. Distribution of coefficient of determination for all detected lipids expressed separately for each lipid class

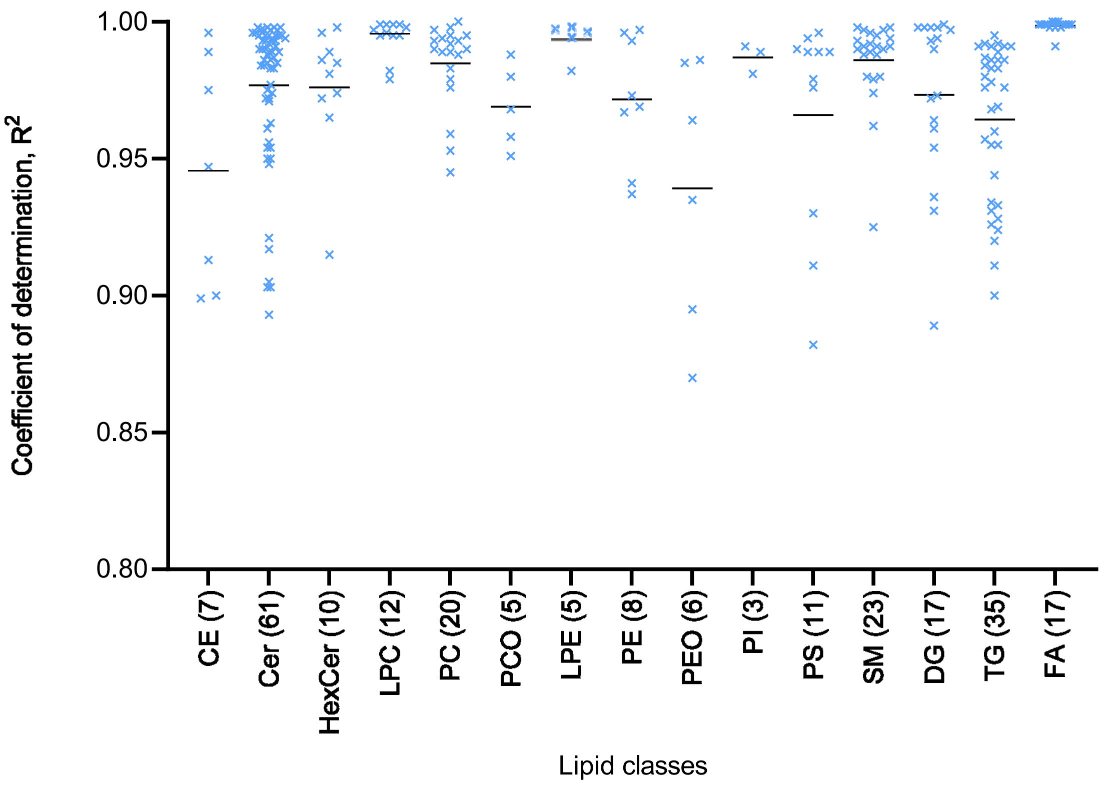

Figure S4. Post-column infusion experiment. QC sample LC-MS runs with post-column injection of 100x diluted SPLASH® LIPIDOMIX® Mass Spec Standard mixture with syringe flow rate of 3 μl/min. Non-scheduled MRM transitions corresponding to each deuterated standard in the mixture were measured to investigate ion suppression or ion enhancement across the time of analysis (8 in positive and 5 in negative mode and the dwell time was set to 30 ms).

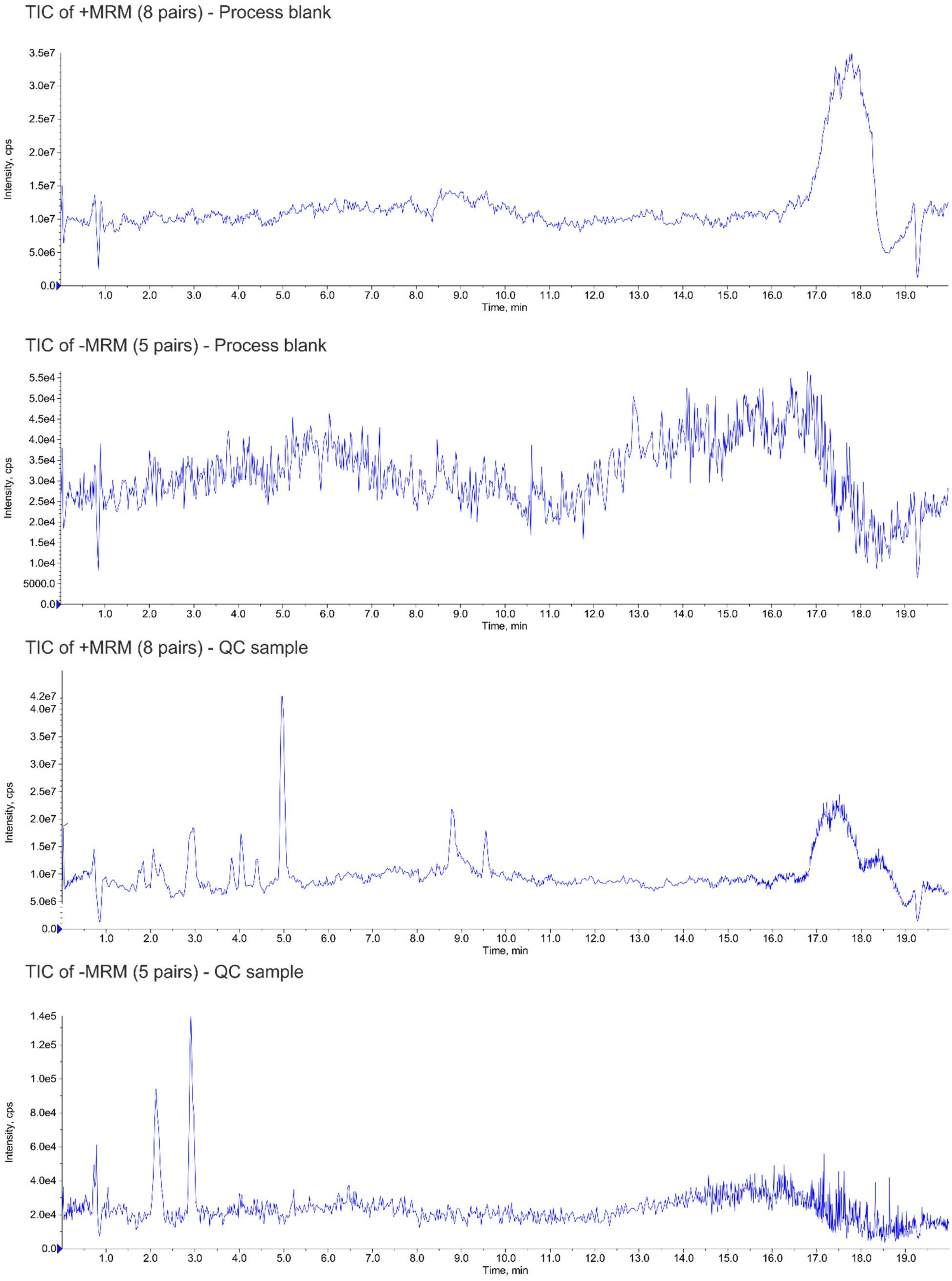

Table S5. Batch layout

Analytical Order	Sample information
1	Extraction solution blank
2	Conditioning QC
3	Conditioning QC
4	Conditioning QC
5	Extraction solution blank
6	Process blank
7	Conditioning QC
8	Conditioning QC
9	Conditioning QC
10	QC1
11	A1L
12	A1R
13	B1L
14	B1R
15	C1L
16	C1R
17	QC2
18	D1L
19	D1R
20	E1L
21	E1R
22	F1L
23	F1R
24	QC3
25	G1L
26	G1R
27	H1L
28	H1R
29	I1L
30	I1R
31	QC4
32	J1L
33	J1R
34	A2L
35	A2R
36	B2L
37	B2R
38	QC5
39	C2L
40	C2R
41	D2L
42	D2R
43	E2L
44	E2R
45	QC6
46	F2L
47	F2R
48	G2L
49	G2R
50	H2L
51	H2R
52	QC7
53	I2L
54	I2R
55	J2L
56	J2R
57	A3L
58	A3R
59	QC8
60	B3L
61	B3R
62	C3L
63	C3R
64	D3L
65	D3R
66	QC9
67	E3L
68	E3R
69	F3L
70	F3R
71	G3L
72	G3R
73	QC10
74	H3L
75	H3R
76	I3L
77	I3R
78	J3L
79	J3R
80	QC11
81	Calibration series 1
82	Calibration series 2
83	Calibration series 3
84	Calibration series 4
85	Calibration series 5
86	Calibration series 6
87	QC12
88	Injection experiment QC13 0.5ul
89	Injection experiment QC14 1ul
90	Injection experiment QC15 1.5ul
91	Injection experiment QC16 2ul
92	Injection experiment QC17 2.5
93	Injection experiment QC18 3ul
94	Extraction solution blank
95	Process blank

Figure S5. QC sample instrument response during analysis (raw data, peak areas divided by average peak area). Red line represents +/- 15 % from 1.

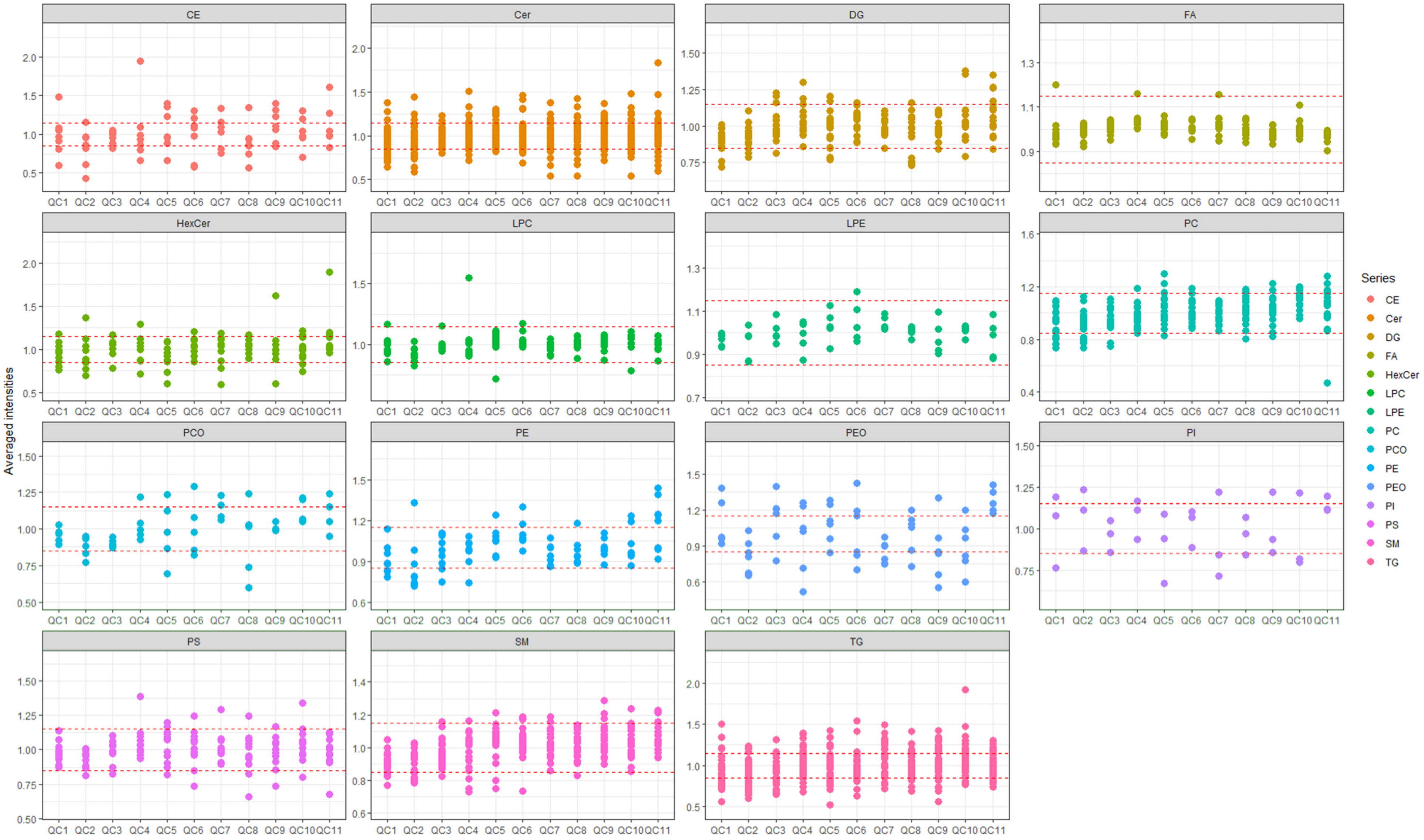

Figure S6. QC sample instrument response during analysis (after LOESS, peak areas divided by average peak area). Red line represents +/- 15 % from 1.

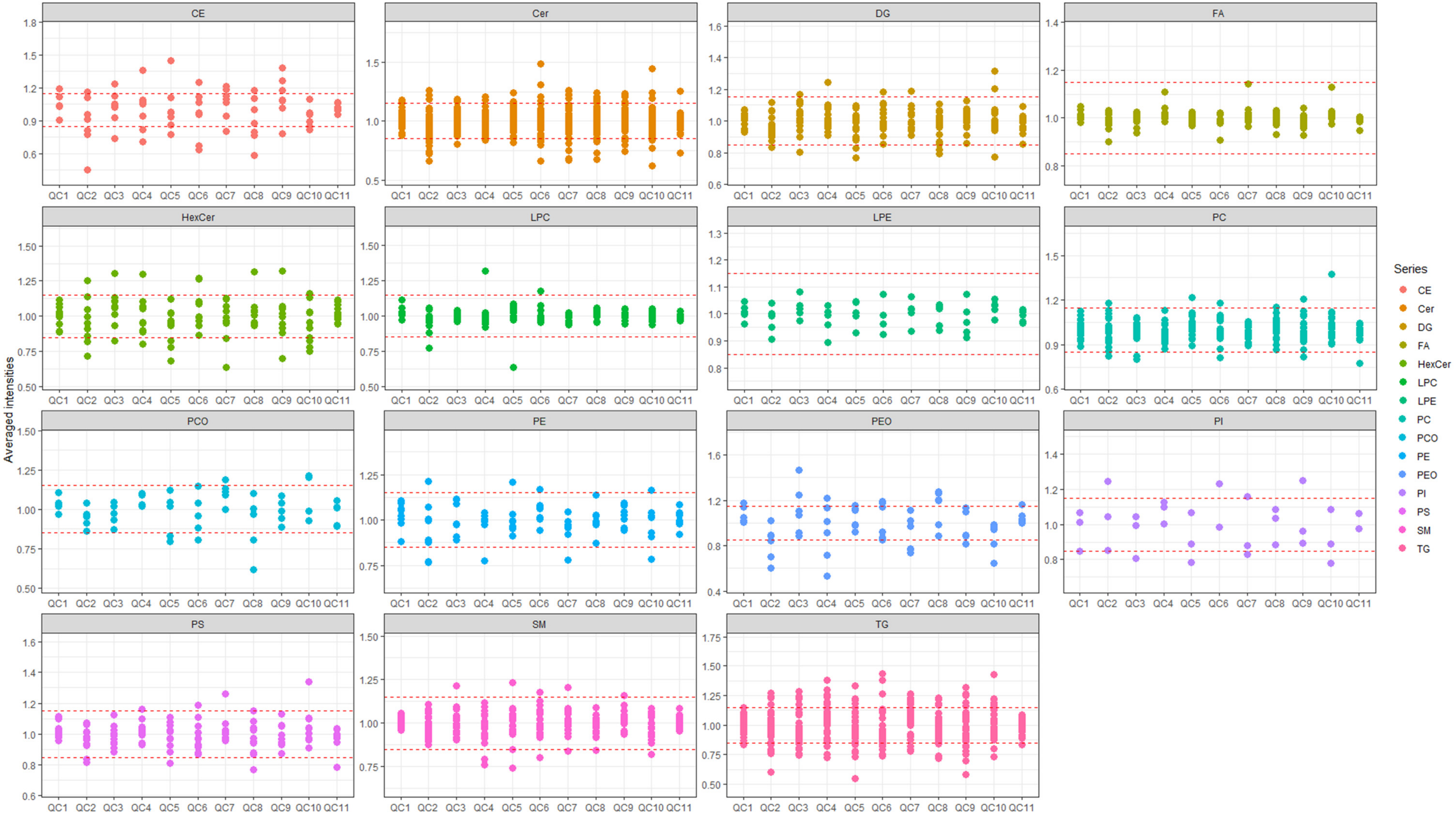

Figure S7. TIC of QCs and samples in both positive and negative mode. Colour of lines represents the final list of 240 identified lipids.

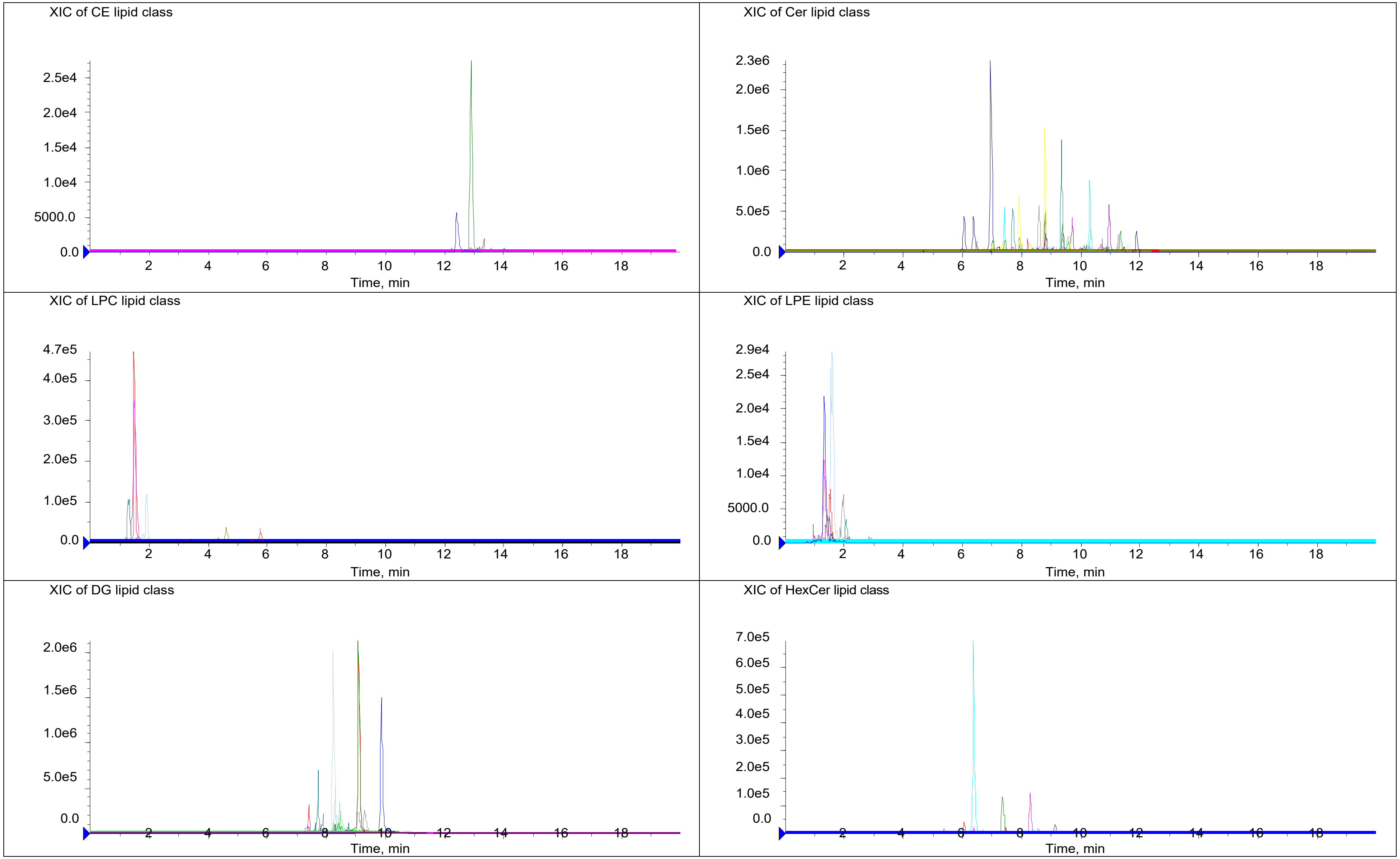

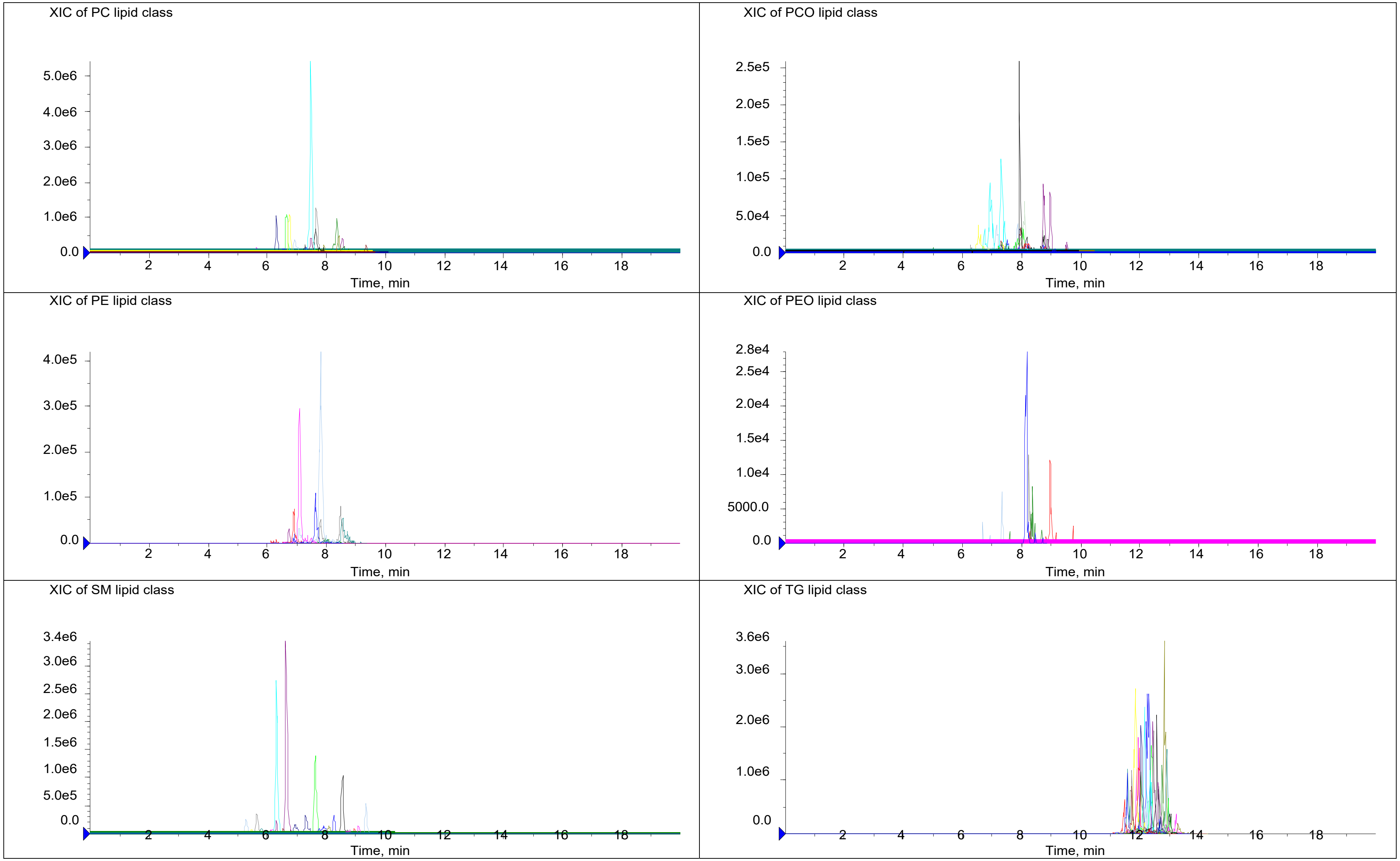
XIC of PC lipid class
5.0e6
4.0e6
3.0e6
2.0e6
1.0e6
0.0
2 4 6 8 10 12 14 16 18
Time, min
XIC of PCO lipid class
2.5e5
2.0e5
1.5e5
1.0e5
5.0e4
0.0
2 4 6 8 10 12 14 16 18
Time, min
XIC of PE lipid class
4.0e5
3.0e5
2.0e5
1.0e5
0.0
2 4 6 8 10 12 14 16 18
Time, min
XIC of PEO lipid class
2.8e4
2.5e4
2.0e4
1.5e4
1.0e4
5000.0
0.0
2 4 6 8 10 12 14 16 18
Time, min
XIC of SM lipid class
3.4e6
3.0e6
2.5e6
2.0e6
1.5e6
1.0e6
5.0e5
0.0
2 4 6 8 10 12 14 16 18
Time, min
XIC of TG lipid class
3.6e6
3.0e6
2.0e6
1.0e6
0.0
2 4 6 8 10 12 14 16 18
Time, min

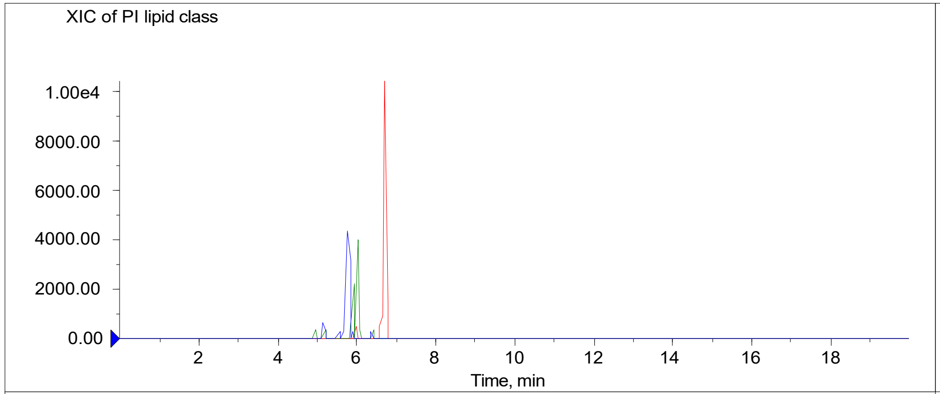
XIC of PI lipid class
1.00e4
8000.00
6000.00
4000.00
2000.00
0.00
2
4
6
8
10
12
14
16
18
Time, min

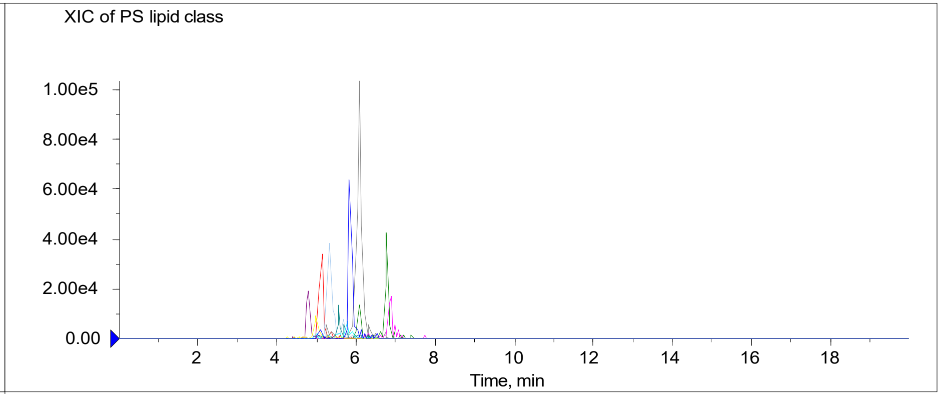
XIC of PS lipid class
1.00e5
8.00e4
6.00e4
4.00e4
2.00e4
0.00
2
4
6
8
10
12
14
16
18
Time, min

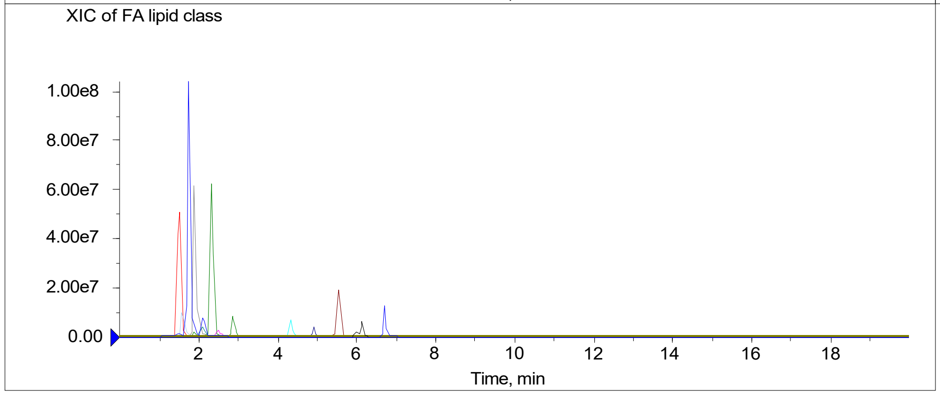
XIC of FA lipid class
1.00e8
8.00e7
6.00e7
4.00e7
2.00e7
0.00
2
4
6
8
10
12
14
16
18
Time, min

Figure S8. XIC of all MRM transitions measured in positive ion mode in QC sample, blank extraction solution (80 % IPA), process blank, 3D filament extract (30s), 3D filament extract (5 minutes).

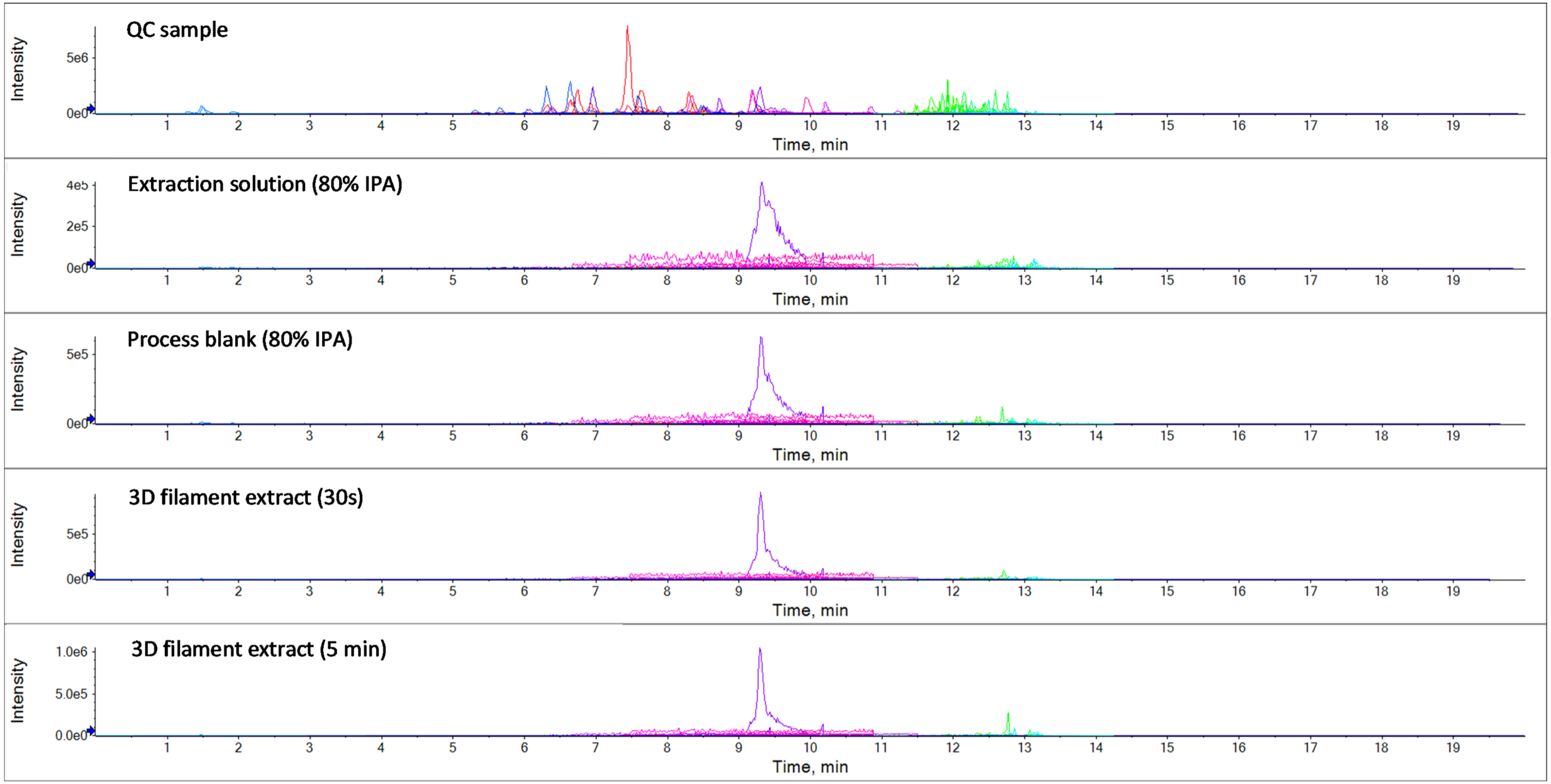

Figure S9. XIC of all MRM transitions measured in negative ion mode in QC sample, blank extraction solution (80 % IPA), process blank, 3D filament extract (30s), 3D filament extract (5 minutes).

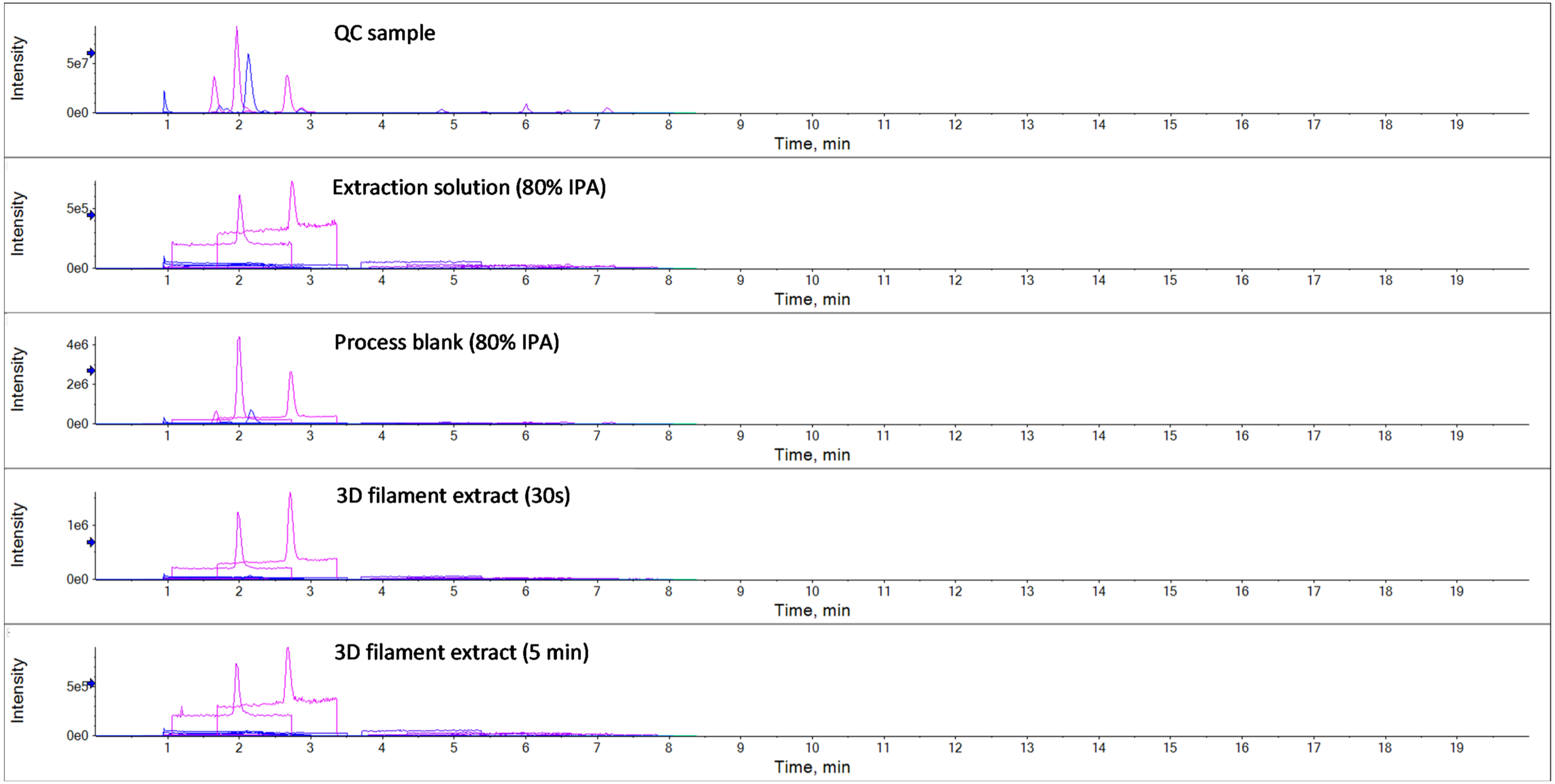

Figure S10. Overview of the variability (CV, %) for 240 detected lipids after ln (natural logarithm) transformation. Quality control samples (QC), intraindi-vidual (CVi), and group variability (CVg). Black lines: medians.

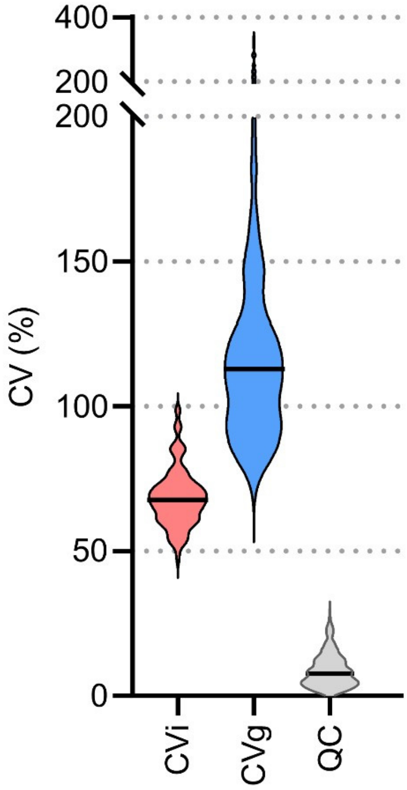

Figure S11. Hierarchical cluster analysis of ln transformed data. Colours represent 10 individuals (A-J) who were sampled from the left (L) and right (R) sides for 3 different days (1-3).

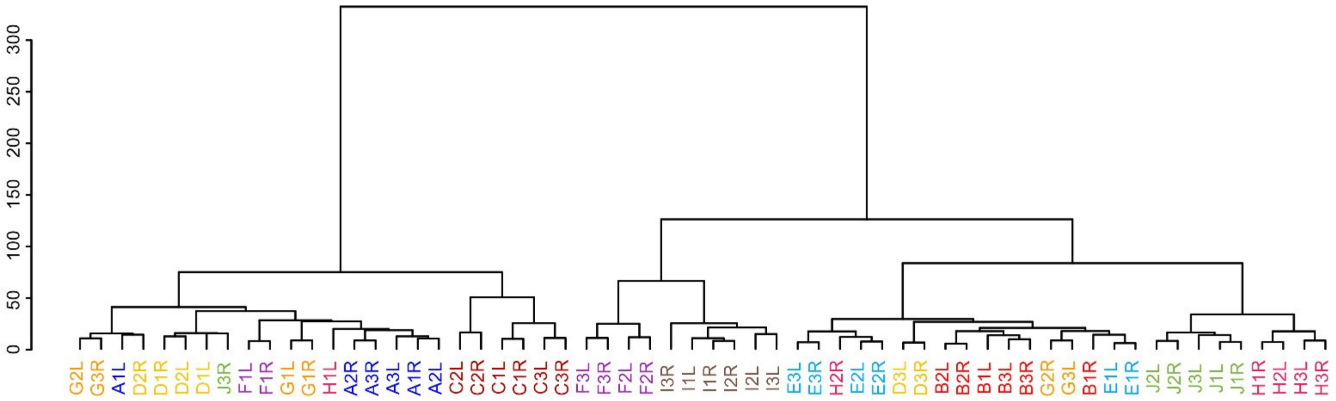

Figure S12. Principal component analysis of the ln transformed data. Different colours represent 10 in-dividuals (A-J).

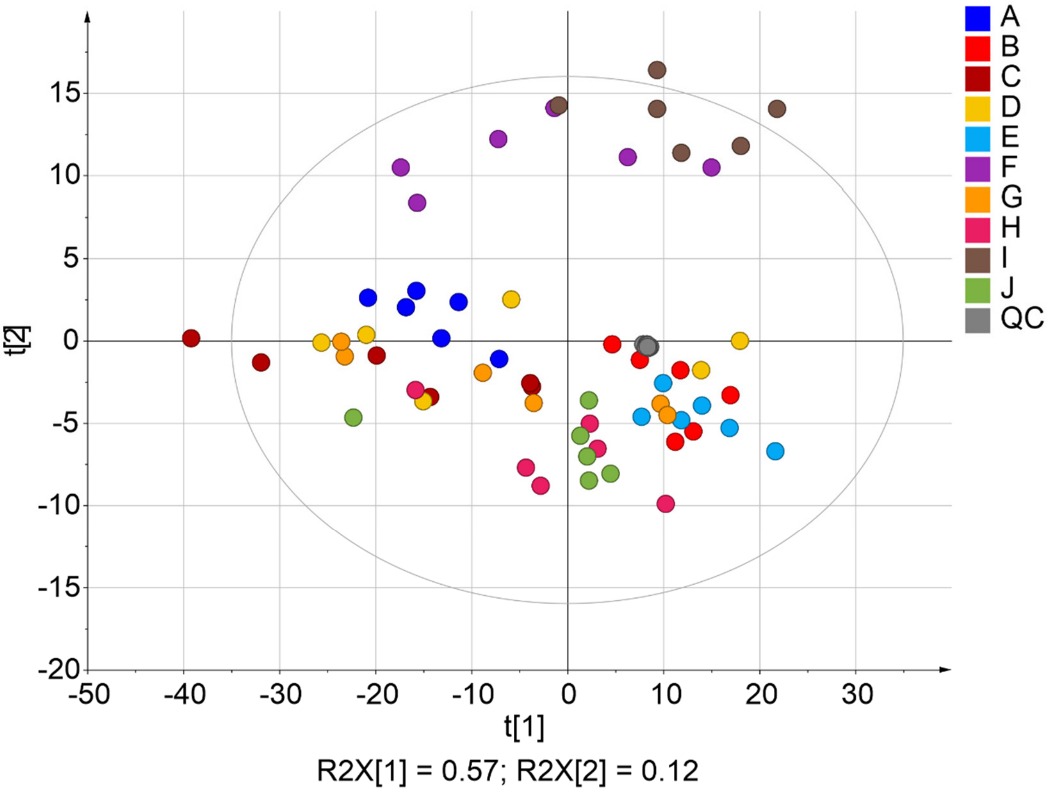

Supplement: Supplementary file 1 [file ijms-22-08054-s001.zip › Supplementary_file_1_SLIDE-Novel_Approach_to_Apocrine_Sweat_Sampling_for_Lipid_Profiling_in_Healthy_Individuals.pdf]
